# Variation in Mutant Prevention Concentrations

**DOI:** 10.3389/fmicb.2019.00042

**Published:** 2019-01-31

**Authors:** Crystal Gianvecchio, Natalie Ann Lozano, Claire Henderson, Pooneh Kalhori, Austin Bullivant, Alondra Valencia, Lauren Su, Gladys Bello, Michele Wong, Emoni Cook, Lakhia Fuller, Jerome B. Neal, Pamela J. Yeh

**Affiliations:** ^1^Department of Ecology and Evolutionary Biology, University of California, Los Angeles, Los Angeles, CA, United States; ^2^Santa Fe Institute, Santa Fe, NM, United States

**Keywords:** antibiotic resistance, selection, *Staphylococcus epidermidis*, repeatability, replication

## Abstract

**Objectives:**Understanding how phenotypic traits vary has been a longstanding goal of evolutionary biologists. When examining antibiotic-resistance in bacteria, it is generally understood that the minimum inhibitory concentration (MIC) has minimal variation specific to each bacterial strain-antibiotic combination. However, there is a less studied resistance trait, the mutant prevention concentration (MPC), which measures the MIC of the most resistant sub-population. Whether and how MPC varies has been poorly understood. Here, we ask a simple, yet important question: How much does the MPC vary, within a single strain-antibiotic association? Using a *Staphylococcus* species and five antibiotics from five different antibiotic classes—ciprofloxacin, doxycycline, gentamicin, nitrofurantoin, and oxacillin—we examined the frequency of resistance for a wide range of concentrations per antibiotic, and measured the repeatability of the MPC, the lowest amount of antibiotic that would ensure no surviving cells in a 10^10^ population of bacteria.

**Results:** We found a wide variation within the MPC and distributions that were rarely normal. When antibiotic resistance evolved, the distribution of the MPC changed, with all distributions becoming wider and some multi-modal.

**Conclusion:** Unlike the MIC, there is high variability in the MPC for a given bacterial strain-antibiotic combination.

## Introduction

The increase in antibiotic-resistant bacteria is globally an urgent public health issue (Dijkshoorn et al., 2007; Nordmann et al., 2007; Davies and Davies, 2010; Brusselaers et al., 2011; Bush et al., 2011; Morehead and Scarbrough, 2018). The minimum inhibitory concentration (MIC), defined as the lowest concentration of an antimicrobial agent that inhibits growth of the wild type population, assuming no mutations, by 99% (Haight and Finland, 1952; Sanders et al., 1984; Sanders, 2001; Obolski et al., 2015) has been used extensively to classify bacteria as resistant to an antibiotic (Dong et al., 1999; Drlica, 2003; Epstein et al., 2004). Yet the MIC is a single measurement of resistance; it captures one parameter of resistance, but not all.

**FIGURE 1:**
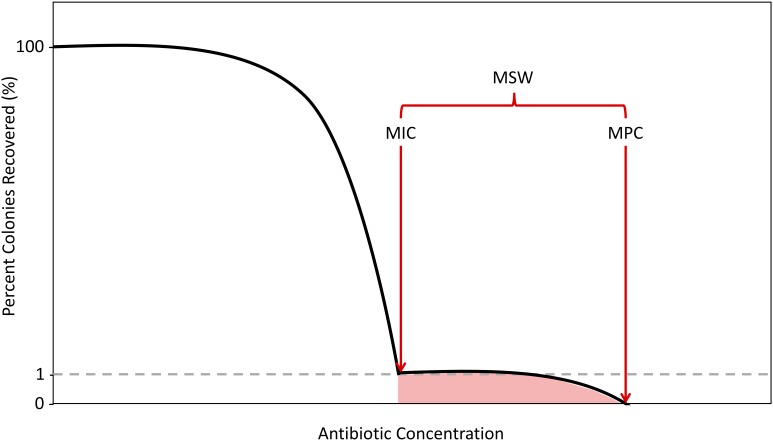
Schematic of the Mutant Selection Window. As antibiotic concentrations increase, the percentage of colonies recovered decreases with two sharp declines demarcating the boundaries of the mutant selection window (MSW), in red. The shaded region selects for single-step resistant mutants. The first decline, which results in a 99% decrease in colonies recovered is determined to be the minimum inhibitory concentration (MIC). The second decline, where there is a 100% decrease in colonies recovered is determined to be the mutation prevention concentration (MPC).

As antibiotic concentrations increase, the first steep decline in colony numbers, representing an ∼1% recovery, corresponds to the MIC. After exposing cells to antibiotics at MIC levels, there will often still exist a population of resistant mutants due to spontaneous mutations, considered to be single-step resistant mutants. As concentrations increase beyond the MIC, these single step mutants will remain until a concentration that reduces colony recovery to 0% is achieved. Above this concentration, no single-step mutants can exist. This concentration is the second metric of resistance, the mutant prevention concentration (MPC). The MPC is defined as the MIC of the least-susceptible, single-step mutant (Dong et al., 1999; Firsov et al., 2003; Allen et al., 2004; Drlica et al., 2006; Hansen et al., 2006; Drlica and Zhao, 2007; Firsov et al., 2008). This is experimentally measured by determining the lowest antibiotic concentration that can kill all single-step resistant mutants within a population size of 10^10^ cells (Feldman, 1976; König et al., 1998; Zhao and Drlica, 2001; Gould and MacKenzie, 2002). This concentration of cells is similar to the numbers of cells found in some infectious cases in clinical situations (Zhao and Drlica, 2001; Gould and MacKenzie, 2002). The concentrations between MIC and MPC, defined as the mutant selection window (MSW), signify the antibiotic concentration range for which evolution of resistance can occur by selecting for the non-susceptible portion of the population (Figure 1; Drlica, 2003; Drlica and Zhao, 2007).

While the MIC for each bacterial antibiotic-strain pair is typically considered a single value with high repeatability (Dong et al., 1999; Zhao and Drlica, 2001; Li et al., 2002; Firsov et al., 2003; Zinner et al., 2003; Allen et al., 2004; Li et al., 2004; Metzler et al., 2004a,b; Marcusson et al., 2005; Hansen et al., 2006; Olofsson et al., 2006; Drlica and Zhao, 2007; Firsov et al., 2008; Liu et al., 2013; Oshima et al., 2017; Zhang et al., 2017), it is unclear if this is true for the MPC. Because the MPC is dependent on the probability and timing of mutations that confer resistance, it seems likely that the MPC would have a greater variance than MICs, but the variation in the MPC has not been well studied.

Previous work typically has examined MPCs using fluoroquinolone antibiotics. Studies using *Staphylococcus aureus* (Dong et al., 1999; Drlica, 2003; Firsov et al., 2003; Allen et al., 2004; Metzler et al., 2004a; Firsov et al., 2008), *Mycobacterium tuberculosis* (Rodriguez et al., 2004; Drlica and Zhao, 2007), and the poultry pathogen *Mycoplasma gallisepcticum* (Zhang et al., 2017) have obtained values for the MPC, and the MSW, by examining the presence of resistant mutants at sub-MPC and MPC antibiotic concentrations *in vitro*. Their results confirm that resistant mutants are enriched when bacteria were exposed to concentrations that fall within the MSW. While the MPC and MSW have been widely described in *M. tuberculosis* in adults as defined values (Rodriguez et al., 2004), in one review of the antibiotic dosing used in child tuberculosis, it was found that the heterogeneity of MICs could result in a range of MPCs (Jaganath et al., 2017). Multiple studies using *Streptococcus pneumonia* (Li et al., 2002; Drlica, 2003; Zinner et al., 2003) and *Haemophilus influenzae* (Li et al., 2004; Metzler et al., 2004b) emphasize the variability in mutation accumulation and observe increasing MSWs with successive mutations. Many studies on the MPC also consider the pharmacokinetics/pharmacodynamics of the antibiotics (Drlica, 2003; Marcusson et al., 2005; Olofsson et al., 2006). Interestingly, one such study found the MIC to be weakly correlated to the MPC using *E. coli* (Marcusson et al., 2005), also suggesting that the MPC may be a more unpredictable resistance parameter. In all of the studies mentioned, it is important to note that there were less than five replicates of the MPC obtained.

Our study focuses on a strain of *Staphylococcus epidermidis*, a gram positive bacterium that colonizes the skin and mucus membranes of the human body, and represents a large part of the normal microflora (Widerström et al., 2012). An opportunistic pathogen, *S. epidermidis* is also the leading cause of infections due to intravenous medical devices, resulting in significant healthcare costs (Uckay et al., 2009). There has been little work done to determine MPC variation using *S. epidermidis*, with one study showing stability in MPC values using two replicate experiments (Liu et al., 2013). Our study uses 20 replicate experiments per bacteria-antibiotic strain to investigate the variability of MPCs. Specifically, we address the following questions: Are the MPCs replicable in highly controlled laboratory conditions? What is the variation in MPCs? Does the variation differ between antibiotics and/or strains? Here we show that the MPC can vary significantly, and the ranges differ between antibiotics and through the evolution of resistance. Our results indicate a large role for stochasticity in determining the MPC of a bacterial strain with a specific antibiotic.

## Materials and Methods

### Culture Conditions

A master tube of *S. epidermidis* (ATCC 14990), was our ancestral strain and grown overnight in Luria Broth (LB) media (10 g tryptone, 5 g yeast extract, and 10 g NaCl), and then frozen with 25% glycerol at -80°C. Several hundred aliquots were made from the master tube and also kept frozen with 25% glycerol at -80°C. *S. epidermidis* (ATCC 14990) was evolved to each of five antibiotics: ciprofloxacin, doxycycline, gentamicin, nitrofurantoin, and oxacillin. We obtained and purified one independent spontaneously resistant mutant for each antibiotic, resulting in five resistant strains. For all resistant strains collected, we confirmed resistance by streak-purifying colonies onto agar plates containing antibiotic concentrations above the known MIC. For all experiments described here, we used freshly thawed aliquots of the ancestral strain and the resistant strains. Each replicate experiment required one aliquot. Strains were grown (aerated) in LB media for approximately 8 h at 37°C to a density of roughly 10^9^ cells per ml and serially diluted to approximately 10^5^ cells per mL for MIC determination on agar plates.

### Antibiotics

We used five antibiotics: Ciprofloxacin hydrochloride (CPR) (MP Biochemicals 199020), Doxycycline hyclate (DOX) (Sigma-Aldrich D9891), Gentamycin sulfate salt (GEN) (Sigma-Aldrich G1264), Nitrofurantoin (NTR) (Sigma-Aldrich N7878), and Oxacillin sodium salt (OX) (Sigma-Aldrich 28221). Ciprofloxacin, a synthetic second-generation fluoroquinolone, inhibits DNA synthesis by inhibiting bacterial enzymes DNA gyrase and topoisomerase, which are involved in the unwinding and supercoiling of DNA during DNA replication (Hooper et al., 1987). Doxycycline, a broad-spectrum tetracycline, inhibits bacterial protein synthesis by binding to the 30S ribosomal subunit and preventing aminoacyl tRNA from binding (Roberts, 1996; Chopra and Roberts, 2001). Gentamicin, an aminoglycoside, inhibits bacterial protein synthesis by targeting the ribosomal A site (Hahn and Sarre, 1969; Yoshizawa et al., 1998). Nitrofurantoin, a multiple-mechanism nitrofuran, inhibits a variety of bacterial enzymes, including those involved in DNA and RNA synthesis as well as carbohydrate synthesis (Shah and Wade, 1989; McOsker and Fitzpatrick, 1994). Oxacillin, a beta-lactam penicillin, inhibits bacterial cell wall synthesis (Park and Strominger, 1957). These antibiotics were chosen because of their clinical importance, widespread use, and different mechanisms of action.

### Determination of Liquid Minimum Inhibitory Concentration (MIC) Estimates

MIC estimates in liquid culture were determined using microtiter plates with serial and equidistant dilutions of antibiotics. Approximately 10^3^–10^4^ cells were inoculated in each well with 100 μl LB and allowed to grow for 22 h, shaken at 220 revolutions per minute (rpm) and incubated at 37°C (Tecan Infinite M200 PRO Multimode Microplate Reader). The liquid MIC estimate was determined by the lowest antibiotic concentration observed to inhibit growth by at least 95%, compared to the positive control. We also included negative controls on each 96 well-plate to validate no contamination of media.

### Determination of Agar MIC

Liquid MIC levels were used as a starting point to determine agar MIC levels. Agar tests tend to yield very similar MIC levels, but on occasion there may be minor differences. We plated two 100 mm agar plates for antibiotic concentrations ranging from 0.2 × liquid MIC and ending at 1.7 × liquid MIC estimate in increments of 0.1 × liquid MIC. Viable cells were quantified as colony forming units (CFUs). We inoculated each plate using 10^5^ cells, resulting in a CFU population that has a limited probability of spontaneous mutation (Martinez and Baquero, 2000; O’neill et al., 2001). These cells were spread via the Copacabana method (Worthington et al., 2001; Mills et al., 2005), which involves the equal distribution of bacteria via sterile glass beads. We conducted the agar MIC assays in duplicate and recorded the median and range for each MIC for each bacterial strain. We prepared agar plates using 1000 mL of MilliQ water, 15 g agar powder, and one 25 g LB tablet (10 g tryptone, 5 g yeast extract, 10 g NaCl, and 1.5 g/L Tris/Tris HCl).

### Determination of Mutant Prevention Concentration (MPC)

MPC was determined as the antibiotic concentration that prevents the growth of any resistant mutants following an inoculum of 10^10^ cells on LB plates containing dilutions of antibiotic (Dong et al., 1999; Drlica, 2003). A population of 10^10^, allows for the consideration of single-step mutants, which is imperative in defining the MPC (Martinez and Baquero, 2000; O’neill et al., 2001). From a frozen aliquot, we grew a bacterial culture overnight for 18 h at 37°C and then inoculated this culture in LB until the inoculum reached an OD_600_ between 0.45 and 0.7. We then centrifuged the bacterial culture (4000 rpm × 4 min, 4°C). We resuspended and combined all bacterial pellets in 7.5 mL of the original supernatant to give 10^10^ cells. We used liquid MIC estimates to plan the incremental concentrations used in MPC experiments. We performed two preliminary MPC experiments with concentrations ranging from 1 × liquid MIC estimate to 64 × liquid MIC estimate, increasing by a factor of two. We repeated MPC experiments 20 times, with three replicates per antibiotic concentration. To measure MPC, we plated at least 10^10^ bacterial cells on agar plates and spread the inoculum via the Copacabana method (Worthington et al., 2001; Mills et al., 2005). Plates were then incubated at 37°C for 72 h. We determined MPC to be the lowest concentration of antibiotic where all three agar plates for a single concentration showed zero colonies. We prepared agar plates using 1000 mL of MilliQ water, 15 g agar powder, and one 25 g LB tablet (10 g tryptone, 5 g yeast extract, 10 g NaCl, and 1.5 g/L Tris/Tris HCl).

### Mutant Selection Window (MSW)

Using MICs and MPCs, we determined the MSWs of ancestral and resistant strains in terms of the MIC of the ancestral strain. Using the MIC of the ancestral strain allowed us to directly compare the MSWs between the two strains.

## Results

**FIGURE 2:**
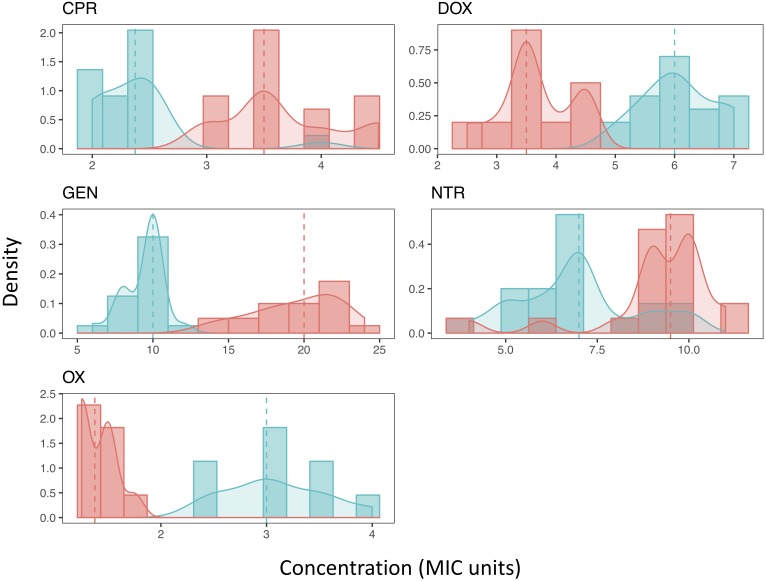
The distribution of Mutant Prevention Concentrations. The MPC distribution of both the ancestral strain (blue) and antibiotic resistant strain (red) for each antibiotic tested. Both the histograms of the data along with the kernel-density estimation is shown. The dashed line represents the median of each sample, respectively. A Shapiro-Wilk test (*p* < 0.05) and Kolmogorov-Smirnov test (*p* < 0.001) both show that all resistant strains distributions cannot be considered normal. Most ancestral strains are also not considered to be a normal distribution (*p* < 0.05), the distribution of the MPCs of the ancestral strain when exposed to either DOX or NTR fail to reject the null hypothesis of a Shapiro-Wilk test (*p* > 0.05). Furthermore, when comparing the MPC distributions between the ancestral strain and the resistant strain for each antibiotic the distributions are not the same (2-sample Kolmogorov-Smirnov test, *p* < 0.001).

We found that MPC estimates varied widely within a single antibiotic, indicating low repeatability of MPC. This was true of most antibiotics tested (Table 1 and Figure 2). The inter-quartile range (IQR) varied among the antibiotics used and whether the strain was the resistant or ancestral strain. The ancestral strain had a more robust signal for a single MPC value where the resistant strain was much more variable (Figure 2).

**Table 1:** Mean, standard deviation, median, and IQR of MPCs for both strains of *Staphylococcus epidermidis* (ancestral and resistant) for all antibiotics tested. All values reported in micrograms per milliliter.

Antibiotic	Strain	Mean	Standard Deviation	Median	IQR
Ciprofloxacin	Ancestral	1.2	0.22	1.2	0.25
	Resistant	4.8	0.68	4.6	0.65
Doxycycline	Ancestral	12.2	1.27	12	2
	Resistant	20.8	3.59	20	3.56
Gentamycin	Ancestral	11	1.54	11.7	2.34
	Resistant	107.3	15.91	110	22
Nitrofurantoin	Ancestral	1	0.25	1.1	0.19
	Resistant	3.2	0.57	3.3	0.35
Oxacillin	Ancestral	24.8	3.81	24	5
	Resistant	47	5.72	46.2	8.4


The distribution of most MPCs do not appear normal (Figure 2). All of the resistant strains did not meet the requirements of a normal distribution (Shapiro-Wilk test (*p* < 0.05) and Kolmogorov-Smirnov test (*p* < 0.001)). The ancestral strains did have a mix of distributions; doxycycline and nitrofurantoin both failed to reject the null hypothesis of a Shapiro-Wilk test (*p* > 0.05). We also demonstrate using a two-sample Kolmogorov–Smirnov test, that the MPC distributions change as resistance evolves. In all direct comparisons of ancestral and resistant strains (with the same antibiotic) the distributions of the MPC values are different (*p* < 0.001).

**FIGURE 3:**
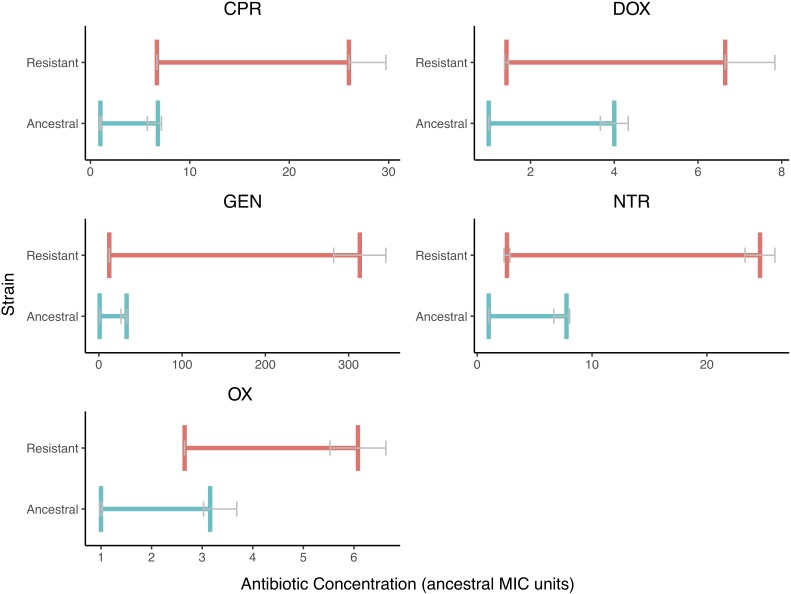
The Mutant Selection Windows of the Ancestral and Resistant Strains. The mutant selection window windows are shown for both resistant (red) and ancestral (blue) strains for each of the five drugs tested. The MIC and MPC shown here are the median value with error bars (IQR) represented in gray. The mutant selection windows for the resist stain are all shifted to higher values and are wider in size. The MPC appears to vary more in the resistant strain than in the ancestral strain.

We also found that the MSW changed when resistance is evolved (Figure 3). There is less variation in the MIC values than there is in the MPC values. The MSW not only shifts but also widens as resistance evolves.

## Discussion

Our results show a range of MPCs in replicate experiments, indicating a large role for stochasticity and limited repeatability for this trait. In this study, the MPC trait is not easily predictable. This variation in MPCs is in contrast to MICs, which are generally predictable for each bacterial strain-antibiotic combination within a particular laboratory setting. For example, although variation in the MIC among different labs has been shown as a result of variations in strains as well as assay variations, individual studies within labs show consistency in the determination of the MIC (Mouton et al., 2017). Thus, while one trait (MIC) is more predictable and repeatable given a certain selection pressure, another (MPC) varies greatly due to stochastic processes. While previous studies indicate that MPCs can be fairly stable (Blondeau et al., 2001; Li et al., 2004; Marcusson et al., 2005; Olofsson et al., 2006), the number of replicates in these studies (two or three), would be insufficient to examine effects of stochasticity on the appearance of mutants.

The change in the MPC is large enough to account for the change of distribution and variation within the resistant strain as there is little to no overlap in the inter-quartile range (IQR). This supports the idea that although the MPC distribution is large and somewhat unpredictable, we can be confident that the MPC of a resistant strain is higher than an ancestral strain.

Our results here suggest two potentially relevant clinical notes. First, it has been proposed that if clinicians target MPCs, there can be no resistant bacteria left in a population within an individual patient (Dong et al., 1999). While this has not proven practical in most cases given the high concentrations of antibiotics needed, there has been work towards determining antibiotic combinations that lower the MPC (Michel et al., 2008). If used clinically (which is entirely hypothetical, since it is not currently used in the clinic), there should be care to understand that MPCs can vary with each bacteria and antibiotic combination and that failure to recognize variation in the MPC could result in inaccurate dosing. Therefore, this study suggests that MPCs should be understood as a range with confidence intervals, rather than as a single number. This study also reveals a significant change in the distribution of the MPC between ancestral and resistant strains, emphasizing the unpredictability of this trait when a bacterial strain acquires a spontaneous mutation conferring antibiotic resistance. Not only do distributions of the MPC in resistant strains increase, but the shapes of the distributions also change considerably. With nitrofurantoin and oxacillin, the distribution of the MPC changes from unimodal distributions in the ancestral strains to bimodal distributions in the resistant strains (See Figure 2). In either case, any intermediate steps taken to move a population off its trajectory towards maximal resistance—for example, using a different antibiotic against a population of bacteria—needs to consider the fact that there may not be a deterministic response of the pathogen population to the new stressor.

There has been some contention as to the utility of MPCs when the resistance mechanisms evaluated *in vitro* do not match the resistance mechanisms that would be found in a clinical setting (Smith et al., 2003). In this study, the acquisition of spontaneous chromosomal mutations was the primary mechanism of resistance when isolating and purifying resistant strains. However, horizontal transfer is typically required for resistance to aminoglycosides like oxacillin, β-lactams like gentamicin, and tetracyclines like doxycycline (Roberts, 1996; Smith et al., 2003). The distributions found in this study offer a first look at the unpredictability of MPC variation in resistant strains. Moreover, ciprofloxacin is a fluoroquinolone in which the mechanism of resistance is largely spontaneous chromosomal mutations (Pantosti et al., 2007).

It is known that the MIC fluctuates with inoculum size, with smaller inocula leading to lower MIC estimates (Granier et al., 2002; Egervärn et al., 2007; Wiegand et al., 2008). Even when testing the MIC values between liquid and agar media, slight differences are found. It would be worthwhile to investigate whether similar fluctuations exist for MPC testing. To elucidate evolutionary potentials in variation, this study used 10^10^ cells, an inoculum size similar to the number of bacterial cells found in naturally-occurring bacterial infections (Feldman, 1976; König et al., 1998; Zhao and Drlica, 2001; Gould and MacKenzie, 2002). Testing a range of large inoculum concentrations may provide further information about how MPCs depend upon cell concentrations present at the time of antibiotic administration. Our findings are particularly relevant to understanding variation in bacterial responses to antibiotics at high cell densities.

Toprak et al. (2012) showed that resistance to different antibiotics involved different types of pathways: some antibiotics had a very stereotyped pathway with similar mutations evolved in the same order, whereas other antibiotics had much more variation in timing and type of mutation (Toprak et al., 2012). With regards to the MPC, it could be illuminating to quantify and examine the specific genetic mutations underlying resistant strains of bacteria at similar and dissimilar MPCs. This would give more information regarding which specific mutations are needed, and how many unique mutations or combinations of mutations exist, to yield high antibiotic resistance. A better understanding of the amount of variation by bacteria and antibiotic could provide a more complete story regarding the variation underlying MPCs. This current study provides a first step, which shows high variability in this important resistance trait.

Luria-Delbruck fluctuations, defined as fluctuations in the frequency of spontaneous mutations in microbial populations (Luria and Delbrück, 1943), may affect the evolutionary trajectory of populations. If a mutation occurs early on in the growth of the population there would be more cells with mutations because of the exponential characteristic of cell division in bacteria (Sarkar, 1991). Conversely, if a mutation arises later, there will be fewer cells exhibiting that mutation. Thus, a low probability event, which occurs early on, may have drastic and amplified results (Skipper, 1983; Rosche and Foster, 2000). Luria-Delbruck fluctuations can, but do not necessarily, have a large impact on the number of resistant mutants in a given population of bacteria (Ford et al., 2013). If a spontaneous mutant arises early in the population growth phase and happens to confer resistance to a given antibiotic, then in the presence of the antibiotic, the ending population will be comprised largely of this resistant mutant and daughter cells. Depending on the exact timing of the appearance of the mutation, a population may exhibit many resistant cells, or very few. Understanding, therefore, the mutations and patterns below the MPC would also be a very useful future study in elucidating fluctuations in the MPC and MSW.

In summary, we find that even in highly controlled laboratory environments, MPCs vary widely, not only from differences in strain and antibiotic, but from replicates with the same strain and same antibiotic. Several other factors may also affect MPC variation, such as CFU concentrations, mutation type, and inocula size and in the future, these factors should be investigated. Understanding how and why the MPC varies can allow us to lay the foundations for more comprehensive dosing strategies that take into consideration the presence and elimination of single-step resistant mutants. From a clinical perspective, caution should be taken when determining how reliable certain therapeutic treatments will be in terms of completely eliminating resistant mutants. From an evolutionary perspective, we show the significant role of stochasticity in bacteria evolving antibiotic resistance.

## Data Availability Statement

The raw data supporting the conclusions of this manuscript will be made available by the authors, without undue reservation, to any qualified researcher.

## Author Contributions

PY conceived of the presented idea and planned the experiments. CG, NL, CH, PK, AB, AV, LS, GB, MW, EC, LF, and JN carried out the experiments. NL analyzed the data. CG, NL, and PY discussed and contributed to the interpretation of results. CG, NL, CH, MW, and PY contributed to the final version of the manuscript. PY supervised the project.

## Conflict of Interest Statement

The authors declare that the research was conducted in the absence of any commercial or financial relationships that could be construed as a potential conflict of interest.
